# Perspectives on MADS-box expression during orchid flower evolution and development

**DOI:** 10.3389/fpls.2013.00377

**Published:** 2013-09-23

**Authors:** Mariana Mondragón-Palomino

**Affiliations:** Department of Cell Biology and Plant Biochemistry, Faculty of Biology and Preclinical Medicine, University of Regensburg, Regensburg, Germany

**Keywords:** Orchidaceae, evo-devo, MADS-box gene, peloric mutant, gene family, transcriptome, model species

## Abstract

The diverse morphology of orchid flowers and their complex, often deceptive strategies to become pollinated have fascinated researchers for a long time. However, it was not until the 20th century that the ontogeny of orchid flowers, the genetic basis of their morphology and the complex phylogeny of Orchidaceae were investigated. In parallel, the improvement of techniques for *in vitro* seed germination and tissue culture, together with studies on biochemistry, physiology, and cytology supported the progress of what is now a highly productive industry of orchid breeding and propagation. In the present century both basic research in orchid flower evo-devo and the interest for generating novel horticultural varieties have driven the characterization of many members of the MADS-box family encoding key regulators of flower development. This perspective summarizes the picture emerging from these studies and discusses the advantages and limitations of the comparative strategy employed so far. I address the growing role of natural and horticultural mutants in these studies and the emergence of several model species in orchid evo-devo and genomics. In this context, I make a plea for an increasingly integrative approach.

## The comparative approach to orchid evo-devo

**Figure 1:**
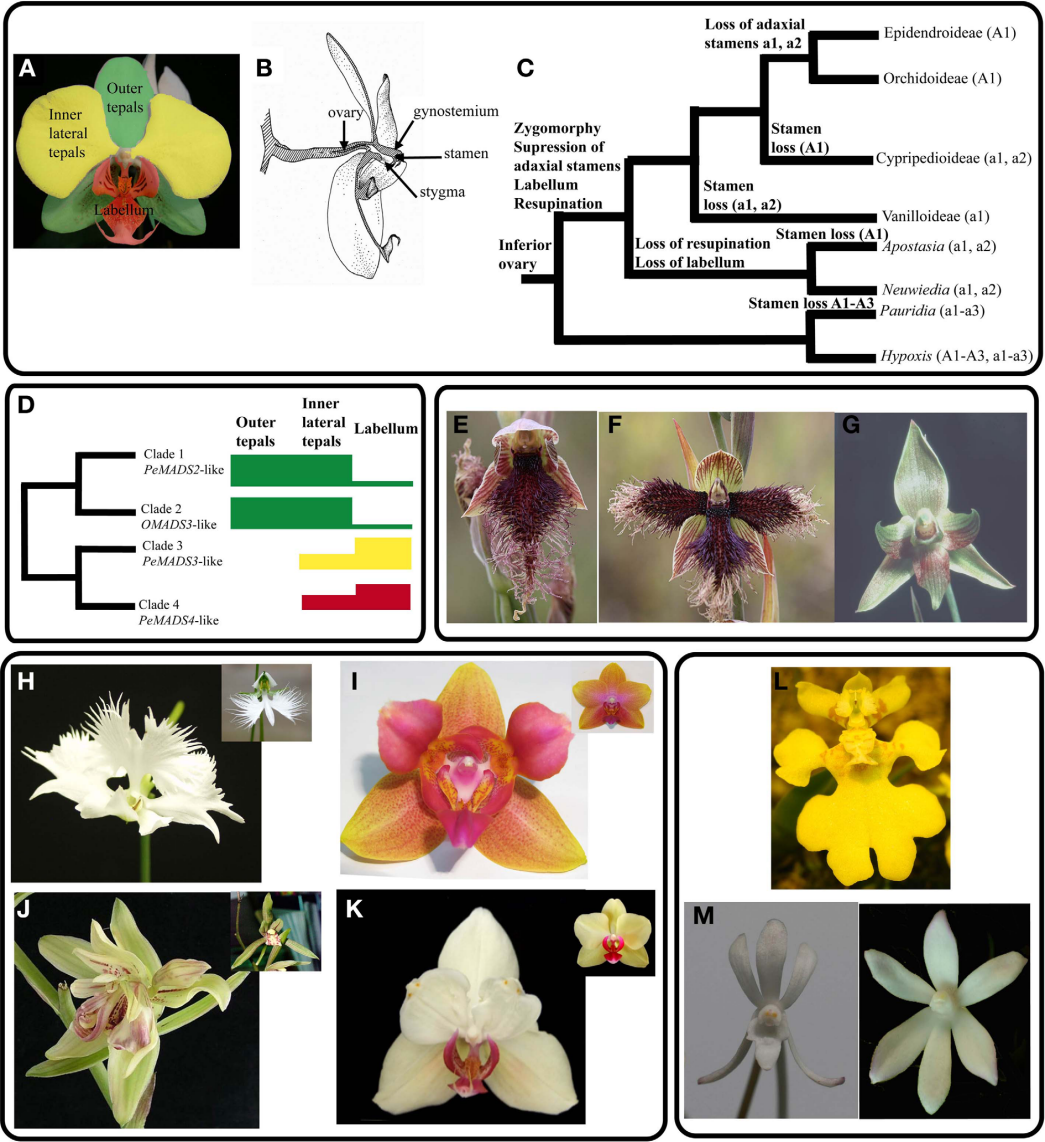
**Wild type and peloric orchids in evo-devo and genomics. (A)** Orchid perianth. The color coding indicates distinct organ identities. **(B)** Reproductive organs. The 180° turn of the pedicel (resupination) together with the opposing position of labellum and stamen enable pollinator attraction as well as precise pollinia removal and placement. **(C)** Perianth and pistils characters gained and lost along the evolution of Orchidaceae from an ancestor with actinomorphic perianth of identical organs and six stamens. *Pauridia* and *Hypoxis* are outgroups. Modified from (Rudall and Bateman, 2002). **(D)** The “orchid code” model associates the phylogenetic relationships of class B *DEFICIENS*-like genes with their differential expression in the perianth and their association to perianth organ identity specification. For example, a higher expression of clade 3 and clade 4 genes is associated to the development of the labellum (red-coded tepal in **A**). **(E)** Wild-type *Calochilus robertsonii*, **(F)** type A peloric *Calochilus robertsonii*, **(G)** type B peloric *Calochilus robertsonii* is recognized as species *Chalochilus imberbis*, **(H)** peloric and wild type (inset) *Habenaria radiata*, **(I)** Type A peloric and wild-type *Phalaenopsis* hyb. “Athens” (inset), **(J)** *Multitepal* mutant and wild-type *Cymbidium ensifolum* (inset), **(K)** *Glyp* mutant and wild-type *Phalaenopsis* “CD” (inset), **(L)** Flower of *Erycina pusilla*, **(M)** Flowers of *Neofinetia falcata* wild-type and Golden Star type B mutant.

The unique diversification of flower morphology in Orchidaceae has taken place in the framework of a relatively conserved structure. Generally orchid flowers consist of three outer tepals similar to each other, two distinct inner lateral tepals and a highly differentiated inner median tepal or labellum (Figure 1A). Female and male reproductive organs are fused into a bilaterally symmetrical (zygomorphic) structure called gynostemium while the ovary is inferior with respect to the rest of the organs (Figure 1B). Along the diversification of this family there have been several major floral morphological transitions: from zygomorphy to actinomorphy, partial to complete suppression of three to five of the original six stamens and the differentiation of the inner median tepal into the distinct labellum (Figure 1C). These transitions and a 180° developmental rotation of the flower pedicel or ovary (resupination) yielded zygomorphic flowers where the abaxially oriented labellum serves pollinators as a landing platform and guide toward the pollinia (Bateman and Rudall, 2006).

Because of the key role of the gynostemium and labellum in orchid reproduction their origin has been a recurring question in botany and evolutionary biology since the 19th century. The finding that flower organ identity is specified by the genetic and physical interaction of MADS domain transcription factors (Bowman et al., 2012) served as a basis in the last 20 years for comparative studies on orchid flower evolutionary development (Table 1). So far the approach employed is essentially based on comparing the expression of orchid MADS-box genes with those of well-characterized model species. This method has generated informative associations between B- and C-like MADS-box genes from orchids with those of *Arabidopsis thaliana* and some non-model species like *Tulipa gesneriana* and *Lilium regale*.

**Table 1:**
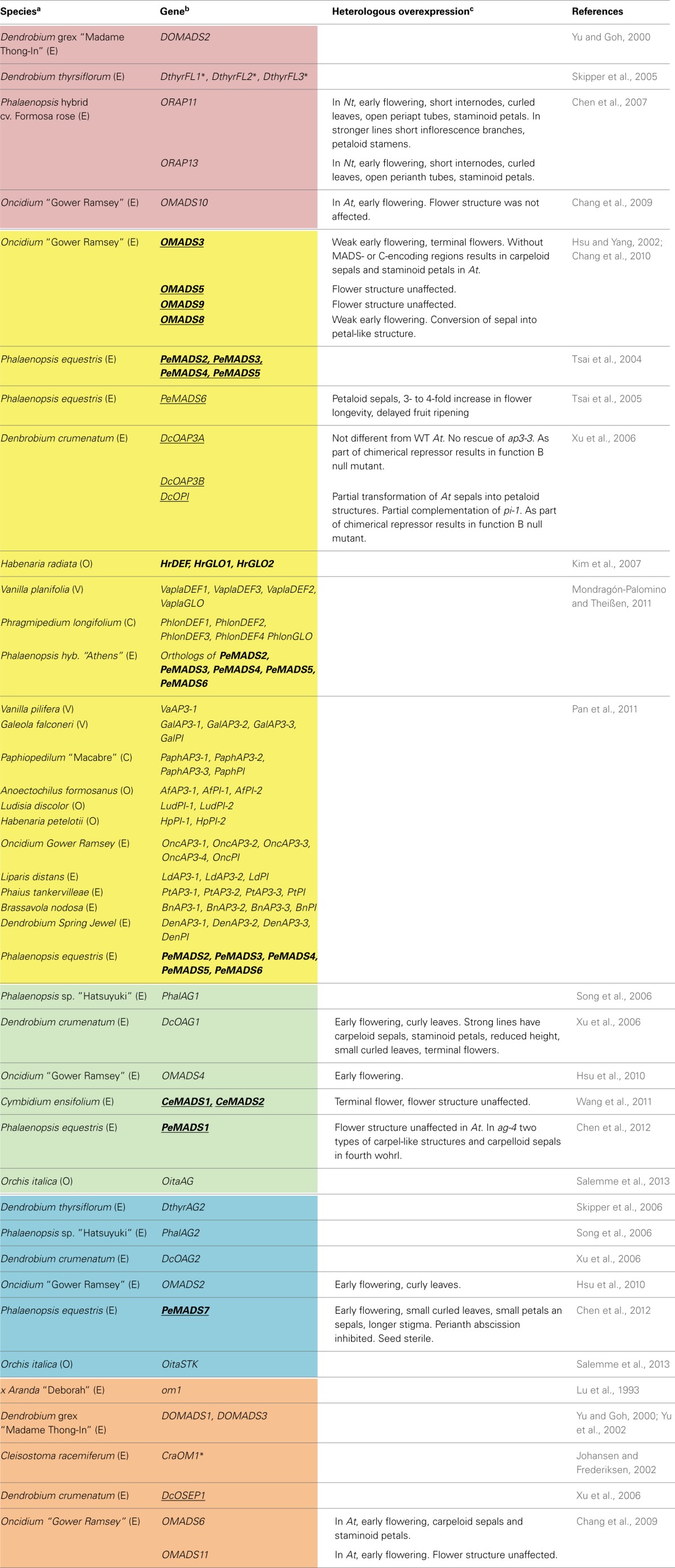
**Class A, B, C, D, and E MADS-box genes characterized in Orchidaceae**.

^a^E, Epidendroidea; O, Orchidoidea; V, Vanilloidea; C, Cypripedioidea.

^b^In bold, Expression analyzed in wild-type and peloric orchids; Underlined, Protein interaction tested.

^c^At, Arabidopsis thaliana; Nt, Nicotiana tabacum.

^*^Expression was measured in complete buds and inflorescences.

The description of the sequence and pattern of expression of the first MADS-box gene isolated from an orchid, *om1* from x *Aranda* “Deborah” (Lu et al., 1993), illustrates the challenges and limitations of strongly relying on knowledge from model organisms. Back in 1993, based on sequence similarity *om1* was considered homologous to *FBP2* from *Petunia hybrida*. However, it was hard to explain the differences on their patterns of expression: while *FBP2* was expressed in petals, stamens, carpels and at a very low level in sepals (Angenent et al., 1992), *om1* was detected in the first and second perianth whorls of x *Aranda* “Deborah.” It was after 2000, when many more MADS-box genes from model species had been characterized and the phylogeny of this family was extensively investigated, that *om1* was confirmed as a *SEPALLATA3*-like gene (Zahn et al., 2005) and expression of *om1* orthologs *DOMADS1* and *DcOSEP1* from *Dendrobium* was confirmed in the perianth as well as in the gynostemium and ovary (Figures 1A,B) (Yu and Goh, 2000; Xu et al., 2006).

In the last 10 years isolation and characterization of individual MADS-box genes from orchids occurred at a faster pace (Table 1). However, because of their role in perianth and stamen specification, nearly all efforts focused on class B and C genes from species in Epidendroideae, the largest orchid subfamily containing most varieties of horticultural importance like *Phalaenopsis*, *Dendrobium* and *Oncidium* (Table 1). The picture emerging from the analysis of A-, C-, D- and E-like MADS-box genes is characterized by several instances of gene duplication in each of these groups, as well as a conserved pattern of expression of each duplicate gene. Specifically, *FRUITFULL*-like genes (class A) are expressed mostly in the gynostemium and in some instances also in the perianth (Yu and Goh, 2000; Skipper et al., 2005; Chen et al., 2007; Chang et al., 2009) while *AGAMOUS*- and *SEEDSTICK*-like genes (class C and D, respectively) are reproducibly expressed in the gynostemium and ovary (Song et al., 2006; Xu et al., 2006; Hsu et al., 2010; Wang et al., 2011; Chen et al., 2012; Salemme et al., 2013). Most of the *SEPALLATA*-like genes (E-like genes) isolated so far are expressed in all flower organs (Lu et al., 1993; Yu and Goh, 2000; Johansen and Frederiksen, 2002; Yu et al., 2002; Xu et al., 2006; Chang et al., 2009).

The orchid family has four ancient, highly conserved lineages of class B genes *DEFICIENS*-like genes (Tsai et al., 2004; Mondragón-Palomino and Theißen, 2008; Mondragón-Palomino et al., 2009). Genes in each of these clades follow a conserved combinatorial pattern of expression associated with the development of specific perianth organs (Figure 1D). Generally *DEF*-like genes in sister clades 1 and 2 are expressed in all flower organs, except the labellum (Chang et al., 2010; Mondragón-Palomino and Theißen, 2011), while genes in sister clades 3 and 4 are expressed in the inner perianth, gynostemium and ovary (Tsai et al., 2004; Xu et al., 2006; Kim et al., 2007; Chang et al., 2010; Mondragón-Palomino and Theißen, 2011; Pan et al., 2011). The specification of outer tepal identity is associated with the combinatorial expression of clade 1 and clade 2 genes, the identity of inner-lateral tepals with the lower expression of clade 3 and clade 4 genes (Figure 1D). Similarly labellum specification depends on the higher expression of clade 3 and clade 4 genes (Mondragón-Palomino and Theißen, 2011). The phylogenetic relationships and patterns of expression of these genes have been integrated in the “orchid code” a model to explain and test the evolution and regulatory relationships of these genes in wild-type and mutant flowers (Mondragón-Palomino and Theißen, 2008, Mondragón-Palomino and Theißen, 2011). The conserved, clade-specific pattern of expression of the four DEF-like genes in representatives from most Orchidaceae subfamilies suggests combinatorial regulation by these genes was established early on the evolution of the Orchidaceae and preserved for millions of years. Generally consistent with the revised “orchid code,” the “HOT model” describes the patterns of expression of class B genes on a temporal and spatial dimension starting from the floral primordial stage (Pan et al., 2011).

In contrast, *GLOBOSA*-like genes the second major lineage of class B genes, are expressed in all flower organs and have not duplicated on a family-wide scale but once in subfamily Orchidaceae (Tsai et al., 2005; Xu et al., 2006; Kim et al., 2007; Mondragón-Palomino et al., 2009; Chang et al., 2010; Mondragón-Palomino and Theißen, 2011; Pan et al., 2011) (Table 1).

ABCDE-class MADS domain transcription factors form dimers and higher order complexes (Egea-Cortines et al., 1999) that bind to CArG-box motifs in regulatory regions of a wide variety of target genes (Kaufmann et al., 2009). The dimeric and multimeric interactions of orchid MADS domain proteins have been described in *Oncidium* “Gower Ramsey,” *Dendrobium crumenatum*, *Phalaenopsis equestris* and *Cymbidium ensifolium* (Hsu and Yang, 2002; Xu et al., 2006; Tsai et al., 2008; Chang et al., 2010; Wang et al., 2011; Chen et al., 2012) (Table 1, underlined gene names).

## Investigating the function of orchid MADS-box genes

### Heterologous expression

Because of technical limitations to transform orchids, most functional analyses of MADS-box class A-, B-, C-, D-, and E-like genes have been performed by means of heterologous ectopic overexpression in *Arabidopsis thaliana* or *Nicotiana tabacum* (Table 1). These experiments employed the strong constitutive promoter CaMV35S and often resulted in early flowering of the species transformed, regardless of which gene was being overexpressed or whether the flower organs are affected or not (Table 1). Early flowering is not exclusively caused by orchid MADS-box genes, as suggested by similar experiments with *Lilium longiflorum LMADS3* (Tzeng et al., 2003). Early flowering and a terminal flower-phenotype are in this context the outcome of activating many other genes, among them other class A, B, C MADS-box genes, which as described for *SEP3*, trigger the flower developmental program (Kaufmann et al., 2009).

Because of the widespread activation capabilities of MADS domain proteins, the effects of heterologous ectopic overexpression are often unpredictable or difficult to attribute to one or few specific genes. For example, in agreement with their role as *GLO*-like genes, the heterologous ectopic overexpression of *DcOPI* and *OMADS8* results in transformation of sepals into petaloid structures and complementation of *pi-1* from *Arabidopsis thaliana* (Xu et al., 2006; Chang et al., 2010). However, it is harder to interpret phenotypes such as a perianth formed by carpelloid sepals and staminoid petals, which result from overexpressing MADS-box genes from different classes like *OMADS3* (*DEF*-like), *DcOAG1* (*AG*-like) or *OMADS6* (*SEP3*-like) (Hsu and Yang, 2002; Xu et al., 2006; Chang et al., 2009) (Table 1). Because this phenotype resembles an *ap2* mutant, it has been proposed that these genes have diverged functionally, however, it is hard to distinguish these claims from the non-specific effects previously mentioned.

Additionally, heterologous overexpression experiments often do not yield phenotypic differences between wild-type and transgenic plants (Table 1). Alternatively, more direct methods for functional characterization are inducible or gene-specific promoters to limit expression to certain tissues and developmental stages.

Advances on the identification and experimental analysis orchid MADS-box promoters have already been made by employing orchid stable transformation to characterize the 5′ regions of *DOMADS1* (Yu et al., 2002). More recently, the transcription factor *OgMYB1* involved in anthocyanin biosynthesis was investigated by means of transient transformation of orchid perianth organs (Chiou and Yeh, 2007). A similar approach has been applied to identify flower-specific promoters in *Oncidium* Gower Ramsey (Hsu et al., 2011b).

### Helpful monsters

Regardless of the underlying genetic or epigenetic causes teratological flowers are phenotypically different from their parental forms. Peloric terata are an special case involving a transition from zygomorphy (bilateral symmetry) to actinomorphy (radial symmetry) (Bateman and DiMichele, 2002; Rudall and Bateman, 2002). Peloric flowers have been reported in wild and cultivated orchids and are classified in type A when the two inner lateral tepals are replaced by labellum-like structures, type B when the labellum is replaced by a inner-lateral tepal (Figures 1E–G) and the more rare type C which involves the substitution of all inner perianth organs by structures resembling outer tepals (Rudall and Bateman, 2002).

Because of the difficulties involved in genetically manipulating orchids, peloric flowers are essential to investigate the developmental pathways specifying location, identity and patterning of each organ in the meristem. Orchid evo-devo has greatly profited from comparing flower ontogeny and patterns of gene expression between wild-type flowers and their peloric forms (Table 1; Figures 1F–M) (Tsai et al., 2004; Kim et al., 2007; Chang et al., 2010; Mondragón-Palomino and Theißen, 2011; Pan et al., 2011; Wang et al., 2011).

The first study comparing the expression of developmental genes in the flower organs of wild-type and type A peloric *Phalaenopsis equestris* suggested the differential expression of *DEF*-like genes *PeMADS2*, *PeMADS3*, *PeMADS4*, and *PeMADS5* is associated with the development of specific flower organs (Tsai et al., 2004). The differential expression of *PeMADS4* in the labellum and in the labellum-like inner lateral tepals of the peloric form suggested this gene is key to the development of this organ (Tsai et al., 2004). Later on, the finding that teratological flowers from *Habenaria radiata* ectopically expressed *HrDEF* (*PeMADS3*-like gene) in the outer perianth was associated to the transformation of outer tepals into organs resembling inner-lateral tepals and labellum, thus suggesting this gene is involved in the specification of the inner perianth (Kim et al., 2007).

A molecular phylogeny of *DEF*-like genes involving representatives from most orchid subfamilies showed that the paralogs first observed in *Phalaenopsis equestris* are actually part of four highly conserved, Orchidaceae-specific clades at least 70 million years old (Mondragón-Palomino and Theißen, 2008; Mondragón-Palomino et al., 2009). On this phylogenetic structure we mapped the expression information available for *PeMADS2*-*PeMADS5*, *HrDEF*, *DcOAP3A*, *DcOAP3B* and *OMADS3* and realized the genes of each clade have a distinct and conserved pattern of expression in the perianth (Mondragón-Palomino and Theißen, 2008). These comparative analyses together with developmental principles derived from the analysis of peloric *Phalaenopsis equestris* (Tsai et al., 2004) and *Habenaria radiata* (Kim et al., 2007) suggested a combinatorial regulatory model for the determination of flower organ identity of orchids. Initially the “orchid code” proposed that specification of labellum identity depended on expression of the clade 4 gene. The “orchid code” was tested and modified accordingly to the results of qRT-PCR analyses on individual flower organs from wild-type and Type A peloric flowers of *Phalaenopsis* hyb. “Athens” (Figures 1H,I), as well as wild-type flowers from *Vanilla planifolia*, *Phragmipedium longifolium*. The comparison between wild-type and peloric flowers showed that in the inner lateral tepals there is a relatively lower amount of both clade 3 and clade 4 genes, while in the labellum and in the labellum-like inner lateral tepals of peloric flowers both genes are expressed at a higher level (Mondragón-Palomino and Theißen, 2011) (Figure 1D).

More recently, the study of floral terata from *Cymbidium ensifolium* and *Phalaenopsis equestris* advanced our understanding of *AGAMOUS*-like genes in orchid flower development. The phenotype of the *multitepal* mutant of *Cymbidium ensifolium* is analogous to *agamous* from *A. thaliana* in that the gynostemium is replaced by an ectopic flower which produces outer and inner tepal-like structures centripetally (Wang et al., 2011) (Figure 1J). Comparison of the patterns of expression of *AGAMOUS*-like genes *CeMADS1* (*DthryAG1*-like) and *CeMADS2* (*DcOAG1*-like) showed that while both genes are expressed in the gynostemium and buds of wild-type flowers, *CeMADS1* is not expressed in developing buds of the *multitepal* mutant. This study thus suggests that *CeMADS1* is a class C gene and both *CeMADS1* and *CeMADS2* are not functionally redundant in the specification of gynostemium identity.

In the *glyp* mutant of *Phalaenopsis* hyb. “CD1” the inner lateral tepals bear ectopic pollinia and their epidermal cells are morphologically intermediate between those of wild-type tepals and those of the gynostemium (Chen et al., 2012) (Figure 1K). The expression of *PeMADS1* (*DthyrAG1*-like, class C) and *PeMADS7* (*DthyrAG2*-like, class D gene) was detected exclusively in the column of the wild-type flowers, while only *PeMADS1* was detected in the gynostemium-like inner lateral tepals of the *glyp* mutant. While this study suggests *PeMADS1* (Chen et al., 2012) might be involved in the development of pollinia and epidermal cells, it is necessary to consider the pattern of expression of the ortholog of *CeMADS2*, the second non-redundant *AGAMOUS*-like gene previously described.

A major question underlying studies with peloric flowers is whether the mutant phenotypes actually result from changes in developmental regulators of flower symmetry determining the location of MADS-box gene expression. An elegant study on the loss of bilateral symmetry of peloric *Linaria vulgaris* flowers as well as analysis of orchid peloria suggest transcription factors from the TCP family could also be at play in the development of this kind of terata (Cubas et al., 1999; Rudall and Bateman, 2002, 2003; Mondragón-Palomino and Theißen, 2009).

## A model for orchid flower evo-devo

The case of *om1* from x *Aranda* “Deborah” discussed in the first section illustrates how the comparative approach to orchid evo-devo is limited by what is known about model species more amenable to genetic analysis and transformation. The recent growth of genomic and transcriptomic resources for Orchidaceae might soon eliminate these barriers.

Several species have been put forward in the literature as candidates or *de facto* model species. Most notably *Phalaenopsis* species and hybrids are frequently employed for the study of orchid development (Table 1) because of their undisputable importance in horticultural breeding and trade (Tang and Chen, 2007) and the availability of horticultural peloric mutants. The genus *Phalaenopsis* is presently the best documented orchid group at the genomic level because the genomes of *Phalaenopsis equestris* (Hsu et al., 2011a) and *Phalaenopsis aphrodite* are being sequenced and genomic resources such as ESTs, transcriptomes and public databases (Su et al., 2013) (http://orchidstra.abrc.sinica.edu.tw/) have been generated for these species (Hsiao et al., 2006; Su et al., 2011; An and Chan, 2012).

Recently transcriptomic resources deposited in the OrchiBase 2.0 (Tsai et al., 2013) (http://orchidbase.itps.ncku.edu.tw) advance ten orchid species as candidate models from each of the five Orchidaceae subfamilies as well as the sister group Hypoxidaceae: *Apostasia shenzhenica* and *Neuwiedia malipoensis* (Apostasioideae); *Vanilla shenzhenica* and *Galeola faberi* (Vanilloideae); *Paphiopedilum armeniacum* and *Cypripedium singchii* (Cypripedioideae); *Habenaria delavayi* and *Hemipilia forrestii* (Orchidoideae); *Cymbidium sinense* as well as the previously mentioned *Phalaenopsis equestris* (Epidendroideae) and *Sinocurculigo taishanica* (Hypoxidaceae) (Tsai et al., 2013). Additionally transcriptomic information for *Dendrobium nobile* and *Oncidium* “Gower Ramsey” is deposited in the databases Orchidstra and *Oncidium* Orchid Genome Base, respectively (Chang et al., 2011) (predictor.nchu.edu.tw).

*Erycina pusilla* (Figure 1L) is an attractive candidate model species because of it can grow rapidly, produce flowers and fruits *in vitro* and has a small genome size (1C = 1.5 pg, *P. equestris* has 1.69 pg and *A. thaliana* has 0.30 pg). Recently, the chloroplast genome of *E. pusilla* and a transcriptome have been sequenced (Pan et al., 2012) (orchidstra.abrc.sinica.edu.tw).

Another promising candidate model species is the wind orchid *Neofinetia falcata* (Figure 1M) because there is a diverse collection of mutant flower morphologies that facilitate systematic analysis of the genes involved in perianth symmetry, spur development and flower organ specification. *N. falcata* grows in laboratory conditions and can be propagated by means of tissue culture and seed germination. Although transcriptomic resources still need to be generated, *Agrobacterium*-mediated transformation, selfing and outcrossing procedures might already enable genetic studies (Duttke et al., 2012).

While the availability of genomic information for a diverse group of species will be a major advance to efficiently isolate and investigate genes involved in orchid flower development, the ability to genetically manipulate orchids stably or transiently is key to directly associate specific genes with their functions. Although stable transformation mediated by *Agrobacterium* (Belarmino and Mii, 2000; Yu et al., 2001) and biolistic bombardment (Chia et al., 1994; Men et al., 2003) have been adapted to *Phalaenopsis*, *Oncidium* and *Dendrobium*, among others, their relatively low efficiency and long regeneration time to obtain flowering plants has limited their application in this area.

Alternatively, virus-induced gene silencing (VIGS) based on *Cymbidium* mosaic virus has been adapted to *Phalaenopsis* (Lu et al., 2006). Although in this study the levels of transcription of *PeMADS6* and other MADS-box genes were significantly affected, flowers developed all their organs regularly, with the exception of greenish streaks on the back of inner and outer tepals (Lu et al., 2006).

## Quo vadis orchid evo-devo?

At its beginnings orchid flower evo-devo greatly profited from knowledge on well- established model species like *Arabidopsis thaliana* and *Antirrhinum majus* as well as from research on other monocot species like *Tulipa gesneriana* and *Lilium regale*. On the other hand, this comparative approach and the technical limitations to genetically manipulate orchids have set important challenges to functionally approach the genetic basis of orchid flower development.

The systematic morphological and molecular characterization of flower terata offers a way around these limitations and has enabled the formulation of several testable models based on the large amount of information on class B MADS-box genes, the most studied developmental genes in this family.

The growing amount of transcriptomic information in a diverse group of orchid species calls for a second wave of integration and comparative analysis at an unprecedented scale. While the apparent number of “endless forms most beautiful,” the sinking prices of RNA-seq and the competitive nature of scientific endeavor might tempt us to sequence “yet another orchid transcriptome” the most significant advances on this subject will come from systematically integrating all available information and testing our findings experimentally by means of unifying developmental and evolutionary hypotheses and models. This process requires not only sharing and comparing information but also the agreement on common concepts for the developmental processes we are investigating. For example, because most studies describe orchid flowers buds based on their size it is not possible to make an objective comparison of transcriptomes or other patterns of gene expression within and between species. An alternative would be that for every species with a transcriptome a description of discrete stages of its development is generated and considered in the design of future expression studies as it is routinely done for *Arabidopsis thaliana* (Smyth et al., 1990; Niederhuth et al., 2013; Takeda et al., 2013).

Because of the prevalent occurrence of gene duplication in orchids the value of gene phylogenies and profiles of gene expression strongly depends on considering as many known duplicates as technically possible. By doing so it is possible to objectively compare studies and minimize the artifacts coming from simultaneously measuring the expression of highly similar paralogs.

Because orchid evo-devo is a relatively young area there are still many major challenges to overcome. In the near future the vitality of its research program depends on the consolidation of one or several model species amenable to genetic manipulation or with a rapid life cycle that enables the fruitful integration of genetic analysis and transcriptomic resources. In the long run, the scientific relevance and reach of orchid evo-devo will rely on its contribution to understanding orchid ecology and evolution in questions like the interaction between environmental variables, pollinators and the activity of developmental transcription factors.

### Conflict of interest statement

The author declares that the research was conducted in the absence of any commercial or financial relationships that could be construed as a potential conflict of interest.
